# Methylation of APC and GSTP1 in Non-Neoplastic Tissue Adjacent to Prostate Tumour and Mortality from Prostate Cancer

**DOI:** 10.1371/journal.pone.0068162

**Published:** 2013-07-09

**Authors:** Lorenzo Richiardi, Valentina Fiano, Chiara Grasso, Daniela Zugna, Luisa Delsedime, Anna Gillio-Tos, Franco Merletti

**Affiliations:** 1 Cancer Epidemiology Unit, Department of Medical Sciences, University of Turin, and CPO-Piemonte, Turin, Italy; 2 Unit of Pathology, Città della Salute e della Scienza di Torino, Turin, Italy; Northwestern University, United States of America

## Abstract

**Background:**

Markers that can discriminate between indolent and aggressive prostate tumours are needed. We studied gene methylation in non-neoplastic tissue adjacent to prostate tumour (NTAT) in association with prostate cancer mortality.

**Methods:**

From two cohorts of consecutive prostate cancer patients diagnosed at one pathology ward in Turin, Italy, we selected 157 patients with available NTAT and followed them up for more than 14 years. We obtained DNA from NTAT in paraffin-embedded prostate tumour tissues and used probe real-time PCR to analyse methylation of the glutathione S-transferase (GSTP1) and adenomatous polyposis coli (APC) gene promoters.

**Results:**

Prevalence of APC and GSTP1 methylation in the NTAT was between 40 and 45%. It was associated with methylation in prostate tumour tissue for the same two genes as well as with a high Gleason score. The hazard ratio (HR) of prostate cancer mortality was 2.38 (95% confidence interval: 1.23–4.61) for APC methylation, and 2.92 (1.49–5.74) for GSTP1 methylation in NTAT. It changed to 1.91 (1.03–3.56) and 1.60 (0.80–3.19) after adjusting for Gleason score and methylation in prostate tumour tissue. Comparison of 2 vs. 0 methylated genes in NTAT revealed a HR of 4.30 (2.00–9.22), which decreased to 2.40 (1.15–5.01) after adjustment. Results were stronger in the first 5 years of follow-up (adjusted HR: 3.29, 95% CI: 1.27–8.52).

**Conclusions:**

Changes in gene methylation are an early event in prostate carcinogenesis and may play a role in cancer progression. Gene methylation in NTAT is a possible prognostic marker to be evaluated in clinical studies.

## Introduction

Prostate cancer is the most common tumour in men in many populations, with a crude incidence of 120–130 per 10^5^ in Europe and the United States [1]. More than 65% of prostate cancers are diagnosed after 70 years of age, and most of these patients will die of competing events. The incidence of prostate cancer has increased dramatically since the introduction of prostate-specific antigen (PSA) testing at the end of the 1980s [1]. Opportunistic PSA-based screening for prostate cancer is widespread, although its ability to decrease mortality is still being debated. The conclusions of a recent review by the U.S. Preventive Services Task Force did not support PSA-based screening as the benefits were not judged to outweigh the harms [2].

The high prevalence of indolent prostate cancer and the use of opportunistic PSA-based screening for prostate cancer may lead to over-treatment. There is presently no consensus on whether radical prostatectomy or active surveillance should be offered as the treatment of choice, especially when it comes to patients with a low risk prostate cancer [3], [4]. It is therefore necessary to better understand the determinants of prostate cancer progression, and to identify prognostic markers for aggressive prostate cancer that can be detected at the time of a first diagnostic biopsy. A number of groups, including ours, have investigated epigenetic changes in prostate tumour tissue in an effort to identify possible prognostic markers [5]–[8]. In particular, several studies have evaluated the presence of CpG island (clusters of dinucleotides of a cytosine and a guanosine) methylation in the promoter of some specific genes in association with biochemical reoccurrence or mortality from prostate cancer. In our previous study, we found that APC (adenomatous polyposis coli) methylation in prostate tumour tissue was associated with a 50% increased risk of mortality from prostate cancer. A few studies support this finding [9]–[12], and others found a prognostic role for methylation in other genes [13], [14]. Methylation of GSTP1 (glutathione S-transferase) promoter is the most extensively investigated epigenetic change in prostate cancer. It can be found in more than 80% of prostate cancers, with a lower prevalence in benign tissue [15]; it is strongly associated with Gleason score but its prognostic role independent of Gleason score is unclear [5].

In this paper we investigated if methylation changes in the non-neoplastic tissue adjacent to prostate tumour (NTAT) are associated with prostate cancer prognosis. Prostate tumours are often multifocal, and a number of studies observed field cancerisation (i.e. alterations in normal tissue adjacent to the tumour) in prostate cancer. Studies comparing NTAT with benign prostate tissue and/or cancer tissue identified alterations in gene expression [16]–[20], telomere DNA content [21], mitochondrial DNA [22], and prevalence of methylation in some genes, including APC [23], GSTP1 [24], [25] and RARβ2 [23], [24]. The mechanisms underlying these alterations are not clearly understood (either extended effects of carcinogens, lateral expansion of the tumour, or influence of the tumour on the adjacent tissue), but field cancerisation likely has clinical relevance. However, few of the previous studies on this issue include follow-up for mortality, thus hampering the evaluation of the prognostic significance of alterations in NTAT. This study focuses on methylation of GSTP1 and APC in the non-neoplastic tissue. It aims at evaluating whether these molecular changes are potential candidates as prognostic markers by evaluating their association with mortality from prostate cancer.

## Patients and Methods

### Study Population

The study is nested in two cohorts of in total 459 consecutive prostate cancer patients who underwent biopsy, radical prostatectomy or transurethral resection of the prostate (TURP) between 1982 and 1988, or between 1993 and 1996, at the pathology ward of the San Giovanni Battista Hospital, Turin, Italy. Details of these two cohorts have been published previously [5]. We obtained DNA from stored paraffin-embedded prostate tumour tissues for all 459 patients. DNA was then analysed for APC, GSTP1 and RUNX3 (Runt-related transcription factor 3) gene methylation using bisulfite modification and methylation-specific PCR [5]. Information on age, source of prostate tumour tissue, residence and tumour grade was available from pathology reports. All original diagnostic slides corresponding to the PETs used for the molecular analyses were traced and re-evaluated by a single uropathologist (LD) in order to assign a uniform Gleason score according to current guidelines. We did not have information on PSA levels or other clinical characteristics. Initially, the two cohorts were followed up until 2007 [5] but, for the present study, we extended the follow-up until August 8, 2010. We used information from death certificates to classify the cause of death as either prostate cancer, or other causes.

Diagnostic slides from patients in the two cohorts were analysed to identify NTAT. We excluded *a-priori* patients from the 1980s whose diagnostic slides included mainly neoplastic tissue and were associated with considerable technical problems in isolating NTAT without contamination from prostate tumour tissue. All other tissue sources, namely TURPs (n = 11) and radical prostatectomies (n = 23) from the 1980s, and biopsies (n = 164), TURPs (n = 45) and radical prostatectomies (n = 34) from the 1990s, were included in the present evaluation. The uropathologist identified and highlighted one area of NTAT on each diagnostic slide. Areas of prostatic intraepithelial neoplasia were excluded. If more than one area of NTAT was present, the pathologist chose the farthest from the tumour. If there was more than one area that met this criterion, the largest area was chosen. The selected portions of NTAT had at least 1.5 mm of distance from tumour cells, in line with previous studies on epigenetic field effect in prostate cancer [23]. This minimal distance was maintained also when the tissue material was relatively poor (i.e. in biopsies); otherwise the patient was excluded from the study. For slides including physically separated tissue fragments (97 patients), both of NTAT and tumour tissue, the in vivo distance between the fragments was unknown, although most likely it was larger than 1.5 mm. For few patients, some of the slides included NTAT only; these slides were included in the study.

At the end of this process, NTAT was identified in 157 patients, all of whom were included in the present study. The selection process from the original 459 patients acted differently depending on the source of the tumour tissue and on the cohort (1980 or 1990 cohorts). Specifically, we selected 91% of the original patients who underwent a TURP or a radical prostatectomy in either of the two cohorts, 33% of those who underwent a biopsy in the 1990 cohort and, consistently with our a-priori criteria, none of those who underwent a biopsy in the 1980 cohort. This selection, being at baseline (i.e. before the occurrence of the outcome), is unlikely to have introduced bias in the associational estimates ([26]).

Four-five (10 µm thick) sequential sections were cut from the paraffin-embedded tissue of the 157 selected diagnostic slides. The slices overlapped exactly with the slides on which the uropathologist highlighted the selected NTAT area. They were manually dissected, removing the portion of NTAT with a disposable thin blade, which was changed between each subject. DNA was then successfully extracted for all study subjects.

The study was approved by the Ethical Committee of the San Giovanni Battista Hospital - CTO/CRF/Maria Adelaide Hospital of Turin (Prot. N. 0021727, March 19th 2007). Anonymized data on gene methylation are available upon request to qualified researchers for the purpose of academic, non-commercial research.

### Molecular Analyses

The commercial QIAamp ® DNA FFPE Tissue Kit (Qiagen, Hilden, Germany) was used for the extraction and purification of genomic DNA. The adequacy of DNA was checked using the PCR amplification of the β-globin housekeeping gene [27].

The genomic DNA samples, including positive controls for methylated and unmethylated status, underwent bisulfite modification using Epitect Bisulfite Kit (Qiagen, Hilden, Germany).

After bisulfite modification, a PCR assay with specific probes to determine the methylation status of APC and GSTP1 promoters was used. This assay allows detection of methylated cytosine with high sensitivity (1 methylated cytosine out of 10,000 unmethylated cytosines [28]). Each PCR reaction contained: 1×PCR buffer, 5.5 µM MgCl 2, 200 µM dNTPs, 600 nM of both primers, 200 nM and 80 nM of specific probe for the methylation of the APC and GSTP1 promoters, respectively, 5 U of Taq, 2 µl of modified DNA with sodium bisulfite and distilled H_2_O to obtain a final volume of 25 µl. The reaction was carried out in a thermocycler (iCycler, BioRad, CA, USA) with the following PCR conditions: 1.30 minutes at 95°C, 15 seconds at 95°C, 1 minute at a primer-specific annealing temperature (60°C) for 50 cycles, and 10 minutes at 72°C. The universal methylated DNA CpGenome (Intergen Co., Oxford, UK) was used as a positive control. Negative controls were included in all reactions.

Primers and probes were chosen based on published sequences (Table 1) [29], [30]. Probes targeted four CpG islands in each gene.

**Table 1:** Primers and probes used for the real-time PCR.

	GENE	
	GSTP1^a^	APC^a^
**PRIMER F**	5′- GATTTGGGAAAGAGGGAAAGGT -3′	5′-GGATTAGGGCGTTTTTTAT-3′
**PRIMER R**	5′- CAAAAAAACGCCCTAAAATC -3′	5′-GTGTGGGCGTACGTGATCGATATGTG-3′
**PROBE**	Fam- TGCGCGGCGATTTCGGG -tamra	Fam-TTCGTCGGGAGTTCGTCGATTG-tamra

a GSTP1, glutathione S-transferase; APC, adenomatous polyposis coli.

Information on APC, GSTP1 and RUNX3 methylation in prostate tumour tissue was already available from our previous study [5]. However, in the previous analyses we used methylation-specific PCR instead of probe real-time PCR. Since the latter method has a higher sensitivity, we retested all prostate tumour tissue samples in which APC or GSTP1 was unmethylated, to make results from prostate tumour tissue and NTAT more comparable. Methylation in RUNX3 was not re-assessed because analyses in the NTAT involved only APC and GSTP1 and the potential additional confounding effect of RUNX3 methylation in the tumour tissue was expected to be limited. Consistently, tumour tissue methylation in RUNX3 has not been considered further in this paper.

### Statistical Analyses

Using multivariable logistic regression, we estimated the prevalence odds ratios of APC or GSTP1 methylation in NTAT for selected characteristics (Gleason score, methylation in prostate tumour tissue).

The effect of NTAT methylation in each of the two genes on cumulative mortality from prostate cancer was investigated taking into account competing risks, and differences in mortality were evaluated with the Gray’s test [31]. We used Cox proportional hazard regression models to estimate the hazard ratio (HR) of mortality from prostate cancer in association with methylation in NTAT. Follow-up duration was used as the time scale and age at diagnosis was introduced as a continuous variable. Both graphical checks and formal tests, based on Schoenfeld residuals (p>0.14), indicated that the proportional hazard assumption was met. APC and GSTP1 methylation in NTAT were analysed in separated models, as well as in a model investigating the number of methylated genes, from 0 to 2. All models were carried out both not adjusting, and adjusting for Gleason score and presence of GSTP1 or APC methylation in prostate tumour tissue.

Since prognostic markers are needed mainly for low- and intermediate-risk tumours, and at the time of diagnosis before deciding about prostatectomy, we conducted two subgroup analyses restricted to: i) study subjects with a Gleason score of 7 or less or ii) subjects who underwent biopsy. We also assessed the association of methylation in NTAT with short-term mortality by conducting separate analyses restricted to the first 5 years of follow-up.

## Results

Selected characteristics of the 157 study subjects are reported in Table 2. Most subjects were diagnosed in the 1990s, with a median survival of 6.79 years. Of the 128 subjects who died during follow-up, 43 men died from prostate cancer. The source of tissue was equally distributed among biopsy, TURP and radical prostatectomy. There was a high prevalence of both APC methylation (82.2%) and GSTP1 methylation (84.1%) in prostate tumour tissue.

**Table 2:** Selected characteristics of the 157 study subjects with prostate cancer.

Characteristic	Number	(%)
**Year of diagnosis**		
1982–1988	28	(17.8%)
1993–1996	129	(82.2%)
**Range survival time (years)**	0.03–24.11	
**Median survival time (years)**	6.79	
**Age at diagnosis (years)**		
40–64	26	(16.6%)
65–69	35	(22.3%)
70–74	40	(25.5%)
≥75	56	(35.7%)
**Mortality**		
Overall	128	(81.5%)
From prostate cancer	43	
From other causes	85	
**Source of tumour tissue**		
Biopsy	54	(34.4%)
TURP^a^	54	(34.4%)
Radical prostectomy	49	(31.2%)
**Gleason score**		
<7	59	(37.6%)
7	41	(26.1%)
≥8	57	(36.3%)
**Methylation in prostate tumour tissue**		
**APC methylation**		
No	28	(17.8%)
Yes	129	(82.2%)
**GSTP1 methylation**		
No	25	(15.9%)
Yes	132	(84.1%)
**Methylation in NTAT** a		
**APC methylation**		
No	88	(56.1%)
Yes	69	(43.9%)
**GSTP1 methylation**		
No	91	(58.0%)
Yes	66	(42.0%)

a TURP, transurethral resection of the prostate;

NTAT, non-neoplastic tissue adjacent to the tumour.

The prevalence methylation in NTAT was 43.9% for APC and 42% for GSTP1. As summarised in Table 3, the pattern of methylation in NTAT correlated with that in prostate tumour tissue: methylation in either of two genes in prostate tumour tissue was associated with methylation of the same gene in NTAT. Gleason score was also an important determinant of both APC and GSTP1 methylation in NTAT.

**Table 3:** Factors associated with gene methylation in non-neoplastic tissue adjacent to the tumour (NTAT).

Factor		APC methylation in NTAT						GSTP1 methylation in NTAT				
		Prevalence(%)		POR^a^	95% CI^a^			Prevalence(%)		POR^a^		95% CI
**APC methylation in prostate tumour tissue**												
No	21.4		1.00			Ref	35.7		1.00		Ref	
Yes	48.8		3.52			1.25–9.92	43.4		1.38		0.55–3.46	
**GSTP1 methylation in prostate tumour tissue**												
No	32.0		1.00			Ref	24.0		1.00		Ref	
Yes	46.2		1.71			0.65–4.46	45.4		2.86		1.00–8.18	
**Gleason score**												
<7	30.5		1.00			Ref	33.9		1.00		Ref	
7	48.8		2.48			1.04–5.92	36.6		1.32		0.53–3.29	
≥8	54.4		3.04			1.32–7.00	54.4		5.10		1.96–13.3	

a POR, prevalence odds ratio adjusted for age (categorised as in Table 1), source of tumour tissue (biopsy, prostatectomy, TURP), calendar year of diagnosis (1980s, 1990s); Gleason score was introduced in the model as alternative to tumour tissue methylation in APC and GSTP1; CI, confidence interval.

**Figure 1:**
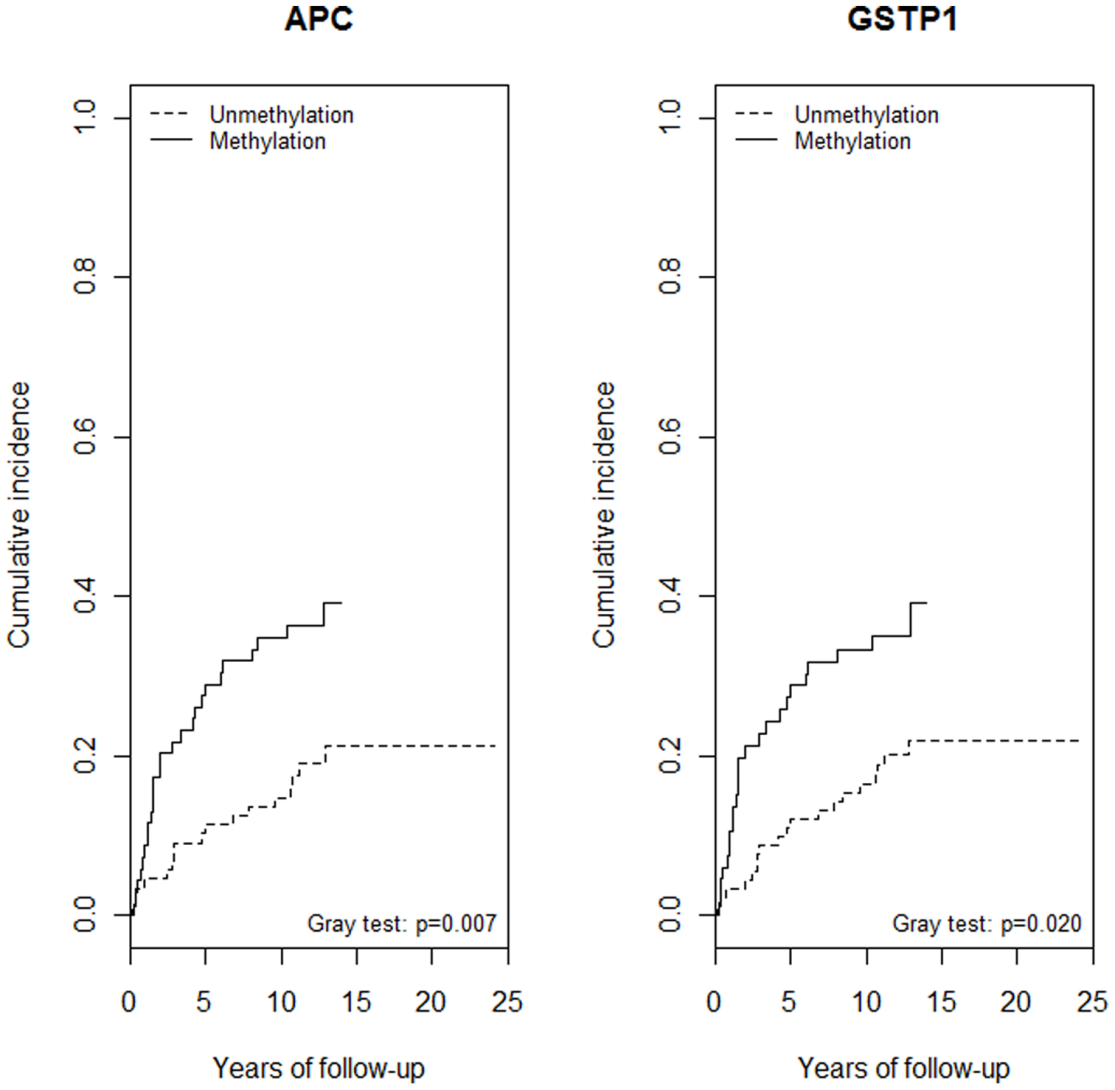
Cumulative mortality from prostate cancer by APC and GSTP1 methylation status in the non-neoplastic tissue adjacent to the tumour.

As shown in Figure 1 NTAT methylation both in APC (Fig. 1a) and GSTP1 (Fig. 1b) were associated with an increased risk of long-term mortality from prostate cancer. Hazard ratios for the presence of methylation were increased by about three-folds (Table 4). These were attenuated after adjustment for methylation in prostate tumour tissue, and after adjustment for Gleason score (HR = 1.91, 95% CI: 1.03–3.56 for APC and HR = 1.60, 95% CI: 0.80–3.19 for GSTP1). Analysis by number of methylated genes in NTAT revealed a positive association with mortality from prostate cancer (p-value for trend = 0.001): the crude HR of mortality from prostate cancer for 2 vs. 0 methylated genes was 4.30 (95% CI: 2.00–9.22) which decreased to 2.40 (95% CI: 1.15–5.01) after adjustment for both gene methylation in prostate tumour tissue and Gleason score (Table 5). Similar associations where found when analyses were restricted to study subjects with Gleason score of 7 or less. We did not find evidence of effect modification introduced by source of tumour tissue (p value = 0.37). Results restricted to subjects who underwent biopsy are reported in Table 5. Table 5 also shows the results for the analyses restricted to the first 5 years of follow-up. The comparison of 2 vs. 0 methylated genes gave a crude HR of 5.71 (95% CI: 2.23–14.6), and 3.29 (95% CI: 1.27–8.52) after adjustment for gene methylation in prostate tumour tissue and Gleason score.

**Table 4:** Hazard ratios (HR) and 95% confidence intervals (CI) of prostate cancer mortality for methylation of APC and GSTP1 in the non-neoplastic tissue adjacent to the tumour (NTAT).

NTAT methylation	N^a^	HR^b^ (95% CI)	HR^c^ (95% CI)	HR^d^ (95% CI)
**APC**				
No	17	1.00	1.00	1.00
Yes	26	2.38 (1.23–4.61)	2.20 (1.15–4.20)	1.91 (1.03–3.56)
**GSTP1**				
No	19	1.00	1.00	1.00
Yes	24	2.92 (1.49–5.74)	2.68 (1.37–5.28)	1.60(0.80–3.19)

a N, number of prostate cancer deaths.

b HR, hazard ratio adjusted for age, calendar year at diagnosis, source of tumour tissue.

c HR, hazard ratio adjusted for age, calendar year at diagnosis, source of tumour tissue, and methylation in tumour tissue.

d HR, hazard ratio adjusted for age, calendar year at diagnosis, source of tumour tissue, methylation in tumour tissue and Gleason score.

**Table 5:** Hazard ratios (HR) and 95% confidence intervals (CI) of prostate cancer mortality for number of methylated genes in the non-neoplastic tissue adjacent to the tumour (NTAT): all study subjects, those with a Gleason score of 7 or less and those who underwent biopsy.

N. of methylated genes	N^a^	HR^b^ (95% CI)		HR^c^ (95% CI)		HR^d^ (95% CI)
		**All patients**				
0	13	1.00	1.00			1.00
1	10	1.13 (0.52–2.45)	1.09 (0.51–2.34)			0.92 (0.42–2.01)
2	20	4.30 (2.00–9.22)	3.74(1.78–7.87)			2.40 (1.15–5.01)
p–value for trend		0.001	0.002			0.032
		**Gleason score of 7 or less**				
0	5	1.00	1.00		1.00	
1	4	1.27 (0.34–4.66)	1.09 (0.33–3.59)		0.76 (0.22–2.66)	
2	5	2.99 (0.63–14.1)	2.14 (0.48–9.53)		2.14 (0.41–11.1)	
p-value for trend		0.195	0.350		0.452	
		**Biopsy cohort**				
0–1^e^	3	1.00	1.00		1.00	
2	12	4.91 (1.39–17.3)	3.44 (0.94–12.67)		2.52 (0.62–10.30)	
		**First 5 years of follow-up**				
0	7	1.00		1.00		1.00
1	7	1.49 (0.53–4.16)		1.47 (0.52–4.15)		1.37 (0.49–3.78)
2	16	5.71 (2.23–14.6)		5.25 (2.12–13.02)		3.29 (1.27–8.52)
p-value for trend		<0.001		0.001		0.019

a N, number of prostate cancer deaths.

b HR, hazard ratio adjusted for age, calendar year at diagnosis, source of tumour tissue.

c HR, hazard ratio adjusted for age, calendar year at diagnosis, source of tumour tissue and methylation in tumour tissue.

d HR, hazard ratio adjusted for age, calendar year at diagnosis, source of tumour tissue, methylation in tumour tissue and Gleason score.

e As there were only three events among subjects with 0 (2 events) or 1 (1 event) methylated gene, these two categories were merged. In addition, number of methylated genes in the tumour tissue and Gleason score were considered as continuous variables.

## Discussion

We found a strong association between presence of APC and GSTP1 methylation in NTAT and long-term mortality and 5-year mortality from prostate cancer. The association remained after adjustment for Gleason score and in analyses restricted to study subjects with a Gleason score of seven or less and in patients who underwent biopsy.

These results support the hypothesis that DNA hypermethylation is an early event in carcinogenesis that can be detected before the tumour becomes morphologically evident; they also support the notion of field cancerisation in prostate cancer and its role in prostate cancer progression. The fact that the pattern of methylation is similar in NTAT and in prostate tumour tissue suggests that the alterations found in these tissues are the consequence of similar mechanisms, including a lateral expansion or influence of the tumour or diffuse effects of the same carcinogens.

We studied methylation in GSTP1 and APC because of their involvement in crucial pivotal cell control pathways. GSTP1 is a “caretaker” gene, preventing genomic damage mediated by carcinogens or oxidants. Defects in “caretaker” genes (i.e. hypermethylation) may favour susceptibility to cancer development. Moreover GSTP1 may interfere with growth or survival signalling pathways by acting as a tumour suppressor gene [32]. Analogously APC is a well characterized tumour suppressor gene, initially identified in colorectal cancer, that plays an integral role in the wnt-signalling pathway and in intercellular adhesion. It interacts with beta-catenin, a protein involved in cellular adhesion and motility. Regulation of beta-catenin prevents genes that stimulate cell division and cell overgrowth [33].

In our study, we used mortality from prostate cancer as the outcome (as opposed to biochemical recurrence) and we had a very long follow-up time. We included both study subjects with a low-risk of prostate cancer, and those with more aggressive prostate cancer. The source of prostate tumour tissue included biopsies, TURPs and radical prostatectomies.

The prevalence of methylation in NTAT that we found is similar or higher to that detected in previous studies, which, however, display considerable inter-study variation [23]–[25], [34], [35]. All these estimates are not directly comparable, as the studies used different protocols for tissue sampling and different molecular techniques. Our molecular methods aimed to maximize sensitivity and we followed a simple protocol to obtain slices of NTAT with reduced risk of contamination from prostate tumour tissue. Although we cannot exclude *a-priori* that contamination did occur in some samples, its frequency was at most, limited, which is demonstrated by the fact that the association between methylation in NTAT and prostate cancer mortality remained practically unchanged after adjustment for methylation in prostate tumour tissue. Had the presence of methylation in NTAT been due to contamination from methylation in prostate tumour tissue, the association between methylation in NTAT and mortality from prostate cancer would have been strongly reduced after adjustment.

The main limitation of our study is the lack of clinical and pathological information other than the Gleason score. In addition we studied a population with, on average, a higher mortality from prostate cancer than typical contemporary patient series. These limitations hamper the possibility of assessing the actual additional prognostic value of methylation in NTAT compared with presently used nomograms. Our study is thus able to suggest a possible role of methytlation in NTAT in prostate cancer progression, although the possibility for clinical translation of this finding requires further investigation in a better defined clinical setting. We also lacked information on treatment at enrolment, implying that our cohort includes a heterogeneous group of patients, which further hampers direct clinical translation of our findings. However, the fact that the source of tumour tissue (biopsy, radical prostatectomy, TURP) was not an effect modifier suggests that our findings on methylation in NTAT are not explained by confounding from patients’ heterogeneities and/or initial treatment.

The magnitude of the association between methylation in NTAT and mortality from prostate cancer that we found is stronger than that usually found for methylation in prostate tumour tissue [5], [8]–[14]. Although the two genes that we studied were selected *a priori* on the basis of substantial evidence of their potential role in prostate carcinogenesis, it is likely that additional prognostic information could be obtained by the study of a larger number of genes.

In conclusion, our findings of frequent APC and GSTP1 methylation in NTAT and their association with mortality from prostate cancer support the notion that changes in gene methylation are an early event in prostate carcinogenesis and play a role in cancer progression. The strength of the association with mortality from prostate cancer and the fact that the association remains in patients with a Gleason score below 8 suggest that the methylation pattern in NTAT could be a possible prognostic marker for prostate cancer to be tested in future clinical studies.
